# Inhibition of Protein Kinase C Delta Attenuates Allergic Airway Inflammation through Suppression of PI3K/Akt/mTOR/HIF-1 Alpha/VEGF Pathway

**DOI:** 10.1371/journal.pone.0081773

**Published:** 2013-11-29

**Authors:** Yun Ho Choi, Guang Yu Jin, Liang chang Li, Guang Hai Yan

**Affiliations:** 1 Department of Anatomy, Medical School, Institute for Medical Sciences, Chonbuk National University, Jeonju, Jeonbuk, Republic of Korea; 2 Yanbian University Hospital, YanJi, China; 3 Department of Anatomy and Histology and Embryology, Yanbian University School of Basic Medical Sciences, YanJi City, Jilin, China; Ludwig-Maximilians-University Munich, Germany

## Abstract

Vascular endothelial growth factor (VEGF) is supposed to contribute to the pathogenesis of allergic airway disease. VEGF expression is regulated by a variety of stimuli such as nitric oxide, growth factors, and hypoxia-inducible factor-1 alpha (HIF-1α). Recently, inhibition of the mammalian target of rapamycin (mTOR) has been shown to alleviate cardinal asthmatic features, including airway hyperresponsiveness, eosinophilic inflammation, and increased vascular permeability in asthma models. Based on these observations, we have investigated whether mTOR is associated with HIF-1α-mediated VEGF expression in allergic asthma. In studies with the mTOR inhibitor rapamycin, we have elucidated the stimulatory role of a mTOR-HIF-1α-VEGF axis in allergic response. Next, the mechanisms by which mTOR is activated to modulate this response have been evaluated. mTOR is known to be regulated by phosphoinositide 3-kinase (PI3K)/Akt or protein kinase C-delta (PKC δ) in various cell types. Consistent with these, our results have revealed that suppression of PKC δ by rottlerin leads to the inhibition of PI3K/Akt activity and the subsequent blockade of a mTOR-HIF-1α-VEGF module, thereby attenuating typical asthmatic attack in a murine model. Thus, the present data indicate that PKC δ is necessary for the modulation of the PI3K/Akt/mTOR signaling cascade, resulting in a tight regulation of HIF-1α activity and VEGF expression. In conclusion, PKC δ may represent a valuable target for innovative therapeutic treatment of allergic airway disease.

## Introduction

Allergic asthma is one of the most common respiratory diseases, and is characterized by chronic eosinophilic airway inflammation, reversible airway obstruction, increased mucus production, and non-specific airway hyperresponsiveness (AHR) [1]. These effects are attributed to T-helper2 (Th2) cells, together with other inflammatory factors, including B cells, mast cells, eosinophils, cytokines, and chemokines. In particular, interleukin (IL)-4, IL-5, and IL-13, which are produced by Th2 cells, are all related to AHR and inflammatory changes in the airway through the activation of eosinophils [2]. Similarly, tumor necrosis factor-alpha (TNF-α) and IL-1β are required for upregulation of eosinophil chemoattractants and adhesion molecules, eosinophilic migration, increase of cytokine release, and enhancement of AHR [3].

Vascular endothelial growth factor (VEGF) is an endothelial cell-specific mitogenic peptide with key roles in angiogenesis and vascular remodeling [4]. Elevated VEGF levels have been observed in tissues and biological samples from individuals with asthma [5]. Moreover, the VEGF level in asthmatic subjects interrelates closely with disease activity, and correlates inversely with the dimension of airway caliber [6]. VEGF-induced peribroncho-vascular angiogenesis is believed to initiate edema and airway narrowing, which further leads to airway vascular remodeling in asthma [4]. Indeed, VEGF might be one of the crucial mediators in allergic airway disease.

Protein kinase C (PKC) is a complex family including three types of isoenzymes. PKC isoforms are classified as classical (α, βI, βII, and γ), novel (δ,ε, θ, and η), and atypical (ζ and τ/λ) [7]. A growing body of research indicates that PKCs play divergent roles in controlling cell growth, differentiation, apoptosis, and carcinogenesis [8]. PKC α, -βI, -βII, -δ, -ε, and –ζ, but not PKC γ, are expressed in human tracheal epithelial cells [9]. Among them, PKC δ potentiates nuclear factor-kappa B (NF-κB) dependent proinflammatory cytokine production in airway epithelial cells, implying the regulatory role of PKC δ in airway inflammation [10]. Subsequently, inhibition of PKC δ activity has been noted to alleviate asthmatic attack by blocking IgE signaling of mast cells in ovalbumin (OVA)-sensitized mice [11]. Additionally, Langlois *et al* has reported that eosinophil migration, which is associated with the pathogenesis of asthma, is impeded by a PKC δ inhibitor [12]. Taken together, PKC δ is suggested to function as a positive regulator of allergic airway response.

The discovery of the drug rapamycin has led to intense study of its target: the mammalian target of rapamycin (mTOR). mTOR is a highly conserved serine/threonine kinase belonging to the family of phosphoinositide 3-kinase (PI3K)-like kinases [13]. mTOR is the master regulator of cell growth and metabolism, predominantly by virtue of controlling the phosphorylation of at least two regulators of protein synthesis: p70 ribosomal S6 kinase (p70S6K) and an inhibitor of translation initiation, eukaryotic initiation factor 4E (eIF4E)-binding protein 1 (4E-BP1) [14]. Thus, dysregulation of this pathway has been implicated in various diseases, including cancer and type 2 diabetes [15]. Rapamycin is used as an immunosuppressant drug to prevent transplant rejection [16]; however, its effects on inflammation in house dust mite (HDM) or OVA-induced models of asthma are mixed [17]-[20]. Nevertheless, these findings provide evidence that mTOR pathway exerts a marked stimulatory effect upon the development of allergic airway disease.

As a major transcriptional activator responsible for cellular response to hypoxia, the hypoxia-inducible factor (HIF)-1 is a heterodimer composed of HIF-1α and HIF-1β subunits. HIF-1β is constitutively expressed in the nucleus and its levels are unaffected by oxygenation conditions, whereas HIF-1α is tightly regulated by O_2_ tension [21]. When cells are exposed to hypoxia, HIF-1α is stabilized and translocated to the nucleus, where it induces VEGF, various tumor growth factors, or apoptosis-related factors [22]. Thus, HIF-1α plays pivotal roles in angiogenesis, vasodilation, and vascular permeability, which may contribute to airway remodeling and inflammation in asthma [23]. Although HIF-1α is primarily accumulated under hypoxic conditions, HIF-1α expression also depends on its rate of *de novo* synthesis. An increasing body of evidence indicates that some growth factors, inflammatory cytokines, and other signaling molecules can stimulate HIF-1α protein synthesis through the activation of mTOR pathway [24].

PI3K, a classical upstream kinase in the mTOR pathway, has been implicated in various immune response and inflammatory processes. The kinase Akt is the main (but not exclusive) intermediate between PI3K and mTOR kinase [25]. As for asthma pathogenesis, numerous studies have indicated that PI3K/Akt modulates AHR, airway inflammation, and vascular permeability through the regulation of VEGF expression mediated by HIF-1α activity [26], [27]. However, irrespective of the importance of PI3K/Akt/HIF-1α/VEGF axis in allergic response, little is known about the functional link among this pathway, mTOR signaling, and PKC δ in this response.

In view of the above findings showing a stimulatory role for VEGF, PKC δ, mTOR, HIF-1α, and PI3K/Akt pathway in the mechanism of allergic inflammatory response, we sought to determine whether there is cross-talk between these stimulatory pathways leading to a tight regulation of AHR and airway inflammation in a murine model of asthma.

## Materials and Methods

### Ethics Statement

All experiments were approved by Institutional Animal Care and Use Committee of Yanbian University School of Medical Sciences, and were in accordance with the Guide for the Care and Use of Laboratory Animals, published by the National Institutes of Health (NIH Publication 82–23, revised 1996) as well as ARRIVE (Animal Research: Reporting In Vivo Experiments) guidelines, produced by the National Centre for the Replacement, Refinement and Reduction of Animals in Research (NC3Rs). The permit number was SCXK(JI)2012-0007. All surgery was performed under sodium pentobarbital anesthesia, and all efforts were made to minimize suffering.

### Animals and experimental protocols

Specific 7-week-old pathogen-free (SPF) inbred female BALB/c mice were purchased from House section of Yanbian University Health Science Center (YanJi, China). Mice were maintained in an animal facility under standard laboratory conditions for 1 week prior to experiments, and provided water and standard chow *ad libitum*. Mice were immunized intraperitoneally with 10 µg of ovalbumin (OVA; chicken egg albumin from Sigma, St. Louis, MO, USA) plus 1.0 mg of aluminum hydroxide adjuvant (Imject® Alum; Pierce, Rockford, IL, USA). A booster injection of 10 µg of OVA plus 1.0 mg aluminum hydroxide adjuvant was given 14 days later. From day 21 to day 23, the immunized mice were challenged by exposure to an aerosol of 1% OVA in phosphate-buffered saline (PBS) for 20 min. The bronchoprovocation was carried out in the vented plastic chamber (18×14×8 cm) adapted for mice. Aerosol particles of approximately 3–5 µm in diameter were created from an ultrasonic nebulizer (NE-U12; Omron, Tokyo, Japan), directed into the plastic chamber, and vented to a fume hood. Each group consisted of five animals. The OVA-sensitized mice treated with saline (SAL) and challenged with aerosolized saline was used as controls (SAL+SAL group). On the other hand, OVA-sensitized mice, which received saline before OVA challenge (OVA+SAL group), belongs to the asthmatic group.

### Administration of rottlerin, LY294002, rapamycin, 2-methoxyestradiol, and CBO-P11

A PKC δ-specific inhibitor, rottlerin [0.3 mg/kg body weight (BW), Sigma], dissolved in dimethyl sulfoxide (DMSO) and diluted with PBS, was injected into the peritoneum of mice 1 and 24 h before the last challenge with OVA. LY294002 (1.5 mg/kg BW, Sigma), dissolved in sterile DMSO-PBS, was administered intratracheally 2 times to each mice, once on day 21 (1 h before the first airway challenge with OVA) and the second time on day 23 (3 h after the last airway challenge with OVA). Rapamycin (4 mg/kg BW, Sigma), dissolved in vehicle (Phosal 50 PG®), was given by oral gavage 10 times at 24-h intervals on days 14 to 23, beginning at 1 h after the booster injection. An inhibitor of HIF-1α, 2-methoxyestradiol (2ME2; 100 mg/kg BW, Sigma), was suspended in 0.5% carboxymethylcellulose (Calbiochem, San Diego, CA, USA) and administered by oral gavage 5 times at 24-h intervals on days 19 to 23, beginning 2 days before the first challenge. The cyclopeptidic vascular endothelial growth inhibitor, CBO-P11, was used to inhibit VEGF activity. CBO-P11 (2 mg/kg BW, Sigma) was administered intraperitoneally 3 times at 24-h intervals, beginning at 1 h before the last inhalation.

### Assessment of airway responsiveness

Airway responsiveness was measured 2 days after the last OVA challenge according to the method of Choi *et al*. [28]. Conscious unrestrained mice were placed in a barometric plethysmographic chamber (All Medicus, Seoul, Korea) and baseline readings were taken and averaged for 3 min. Aerosolized methacholine (Mch) in increasing concentrations (from 2.5 to 50 mg/ml) was then nebulized through an inlet of the main chamber for 3 min, and readings were taken and averaged for 3 min after each nebulization. The bronchopulmonary resistances are expressed as enhanced pauses (Penh), which were calculated as: [expiratory time (Te)/relaxation time (RT) – 1] × [peak expiratory flow (PEF)/peak inspiratory flow (PIF)], according to the manufacturer's protocol. The results are expressed as the percentage increase in Penh over the baseline, following challenge performed with each concentration of Mch, where the baseline Penh (after PBS challenge) is expressed as 100%.

### Collection of bronchoalveolar lavage (BAL) fluid and differential cell count

Immediately following assessment of airway responsiveness, mice were anesthetized and the tracheas were cannulated while gently massaging the thorax. The lungs were lavaged with 0.7 ml of PBS. The BAL fluid samples were collected and the number of total cells in a 0.05 ml aliquot was counted using a hemocytometer (Baxter Diagnostics, Deerfield, IL, USA). The remaining samples were centrifuged, and the supernatants were stored at –70°C until need for the assay of the levels of total and OVA-specific IgE, IL-4, IL-5, IL-13, TNF-α, IL-1β, eotaxin, intercellular adhesion molecule-1 (ICAM-1), vascular cell adhesion molecule-1 (VCAM-1), and VEGF. The cell pellets were resuspended in PBS and cytospin preparations of the BAL cells were stained with Diff-Quik solution (International Reagents, Kobe, Japan). The cell differentials were then enumerated based on the cell morphology and staining profile. The counting was done by an observer blind to the experimental treatments.

### Measurement of plasma exudation

To assess lung permeability, Evans blue dye (EBD) assay was performed as described previously [26]. Briefly, EBD was dissolved in 0.9% saline at a final concentration of 5 mg/ml to assess lung permeability. Mice were weighed and injected with 20 mg/kg EBD in the tail vein. After 30 min, mice were killed, and their chests were opened. PBS containing 5 mmol/l EDTA was perfused through the aorta until all venous fluid returning to the opened right atrium was clear. Lungs were removed and weighed wet, followed by extraction of extravasated EBD by incubation of biopsies in 1 ml formamide at 55°C for 24 h and measurement of absorbance at 620 nm using a spectrophotometer (Spectra MAX PLUS, Molecular Devices, Sunnyvale, CA, USA). The dye extracted was quantified by interpolation on a standard curve of dye concentrations in the range of 0.01 to 10 µg/ml and is expressed as nanograms of dye per milligram of wet lung.

### Measurement of the levels of IgE, cytokines, eotaxin, adhesion molecules, and VEGF

The levels of total and OVA-specific IgE, IL-4, IL-5, IL-13, TNF-α, IL-1β, eotaxin, ICAM-1, VCAM-1, and VEGF in BAL fluids were determined using the mouse enzyme-linked immunosorbent assay (ELISA) kits (R&D Systems, Minneapolis, MN, USA), as the manufacturer's instructions. The sensitivity for each cytokine is 2.0 pg/ml for IL-4, IL-5, IL-13, TNF-α, IL-1β, and VEGF. The lower limits of detection for ICAM-1 and VCAM-1 are 1.5 pg/ml and 2 ng/ml, respectively.

### Histological examination of murine lung tissue

Lungs were fixed with 10% formalin, and the tissues were embedded in paraffin. Fixed tissues were cut at 4 µm, placed on glass sides, and deparaffinized. Sections were stained with hematoxylin-eosin and periodic acid-Schiff (PAS) for light microscopic examinations. The degree of cell infiltration in the airway was scored in a double-blind screen by two independent investigators. As described elsewhere [29], the degree of peribronchial and perivascular inflammation was evaluated by using a scoring of 0–3, 0, little or no detectable inflammation; 1, occasional cuffing with inflammatory cells; 2, most bronchi or vessels surrounded by a thin layer (1-5 cells) of inflammatory cells; 3, most bronchi or vessels surrounded by a thick layer (>5 cells) of inflammatory cells. For immunohistochemistry of mucin 5AC (Muc5AC), VEGF, and phosphorylated (p)-mTOR, the deparaffinized sections were incubated with a primary antibody against Muc5AC, VEGF, or p-mTOR, followed by reaction with peroxidase-conjugated secondary antibodies. After immunostaining, the slides were counterstained with hematoxylin and then mounted with an aqueous mounting medium (Thermo Shandon Immu-Mount, Pittsburgh, PA, USA) and photomicrographed (Eclipse E600, Nikon, Japan).

### Western blot analysis

As described previously [26], [30], freshly isolated lung tissues, whole cells, or nuclear extracts were homogenized and prepared in the presence of protease inhibitors, and protein concentrations were determined using the Bradford reagent (Bio-Rad, Hercules, CA, USA). A 30 µg sample of protein from the lung homogenates was loaded per lane on a 12% SDS-PAGE gel. Electrophoresis was then performed. The proteins were then transferred to nitrocellulose membranes. Western blot analysis was performed using the polyclonal antibodies against IL-4, IL-5, IL-13, eotaxin (R&D Systems), chloride channel, calcium activated, family member 3 (CLCA3), Muc5AC (Abcam, Cambridge, MA, USA), TNF-α, IL-1β, ICAM-1, VCAM-1, VEGF, actin (Santa Cruz Biochemicals, Santa Cruz, CA, USA), p-mTOR, mTOR, p-p70S6K, p70S6K, p-PI3K, PI3K, p-Akt, Akt, p-PKC δ, or PKC δ (Cell Signaling, Beverly, MA, USA). Besides, the levels of HIF-1α and HIF-1β were analyzed with monoclonal antibodies against these proteins (Abcam). The binding of all the antibodies was detected using an ECL detection system (iNtRON Biotechnology, Seoul, Korea), according to the manufacturer's instructions.

### Cell culture

The human bronchial epithelial cell line BEAS-2B cells were purchased from American Type Culture Collection (Rockville, MD, USA). The cells were grown and maintained as previously described [31]. We pretreated these cells for 6 h with 4 µM rottlerin. For TNF-α/IL-4 stimulation, the medium was replaced, and the cells were cultured in medium containing 50 ng/ml each cytokine as mentioned [32] for up to 24 h. None of the agents used significantly affected cell morphology or viability under these conditions.

### RNA interference experiments

The small interference RNA (siRNA) oligonucleotide for PKC δ was obtained from Santa Cruz Biochemicals. Transfection of siRNA was performed at a concentration of 100 nM using Lipofectamine 2000, as described elsewhere [33]. As a control for siRNA, we used a corresponding random siRNA sequence (siRNA-Con: sense 5′−AGU UCA ACG ACC AGU AGU CTT−3′ and antisense 5′−GAC UAC UGG UCG UUG ATT−3′). Control cells were transfected without oligonucleotides under the same conditions. After transfection, the cells were split in aliquots, grown in six-well dishes for 24 h, and then exposed to indicated condition for preset time periods. Cells were lysed, and total proteins were extracted as described above.

### Densitometric analysis and statistical analysis

All immunoreactive and phosphorylation signals were analyzed by densitometric scanning (Gel Doc XR; Bio-Rad, Hercules, CA, USA). Data are expressed as mean ± SEM. Statistical evaluation of the data was performed using ANOVA, followed by Dunnett's *post-hoc* test, employing Prism 5 software (GraphPad Software, San Diego, CA, USA). Results with *p*<0.05 were considered statistically significant.

## Results

### Effect of rottlerin, LY294002, rapamycin, 2ME2, or CBO-P11on cellular changes in BAL fluids

**Figure 1:**
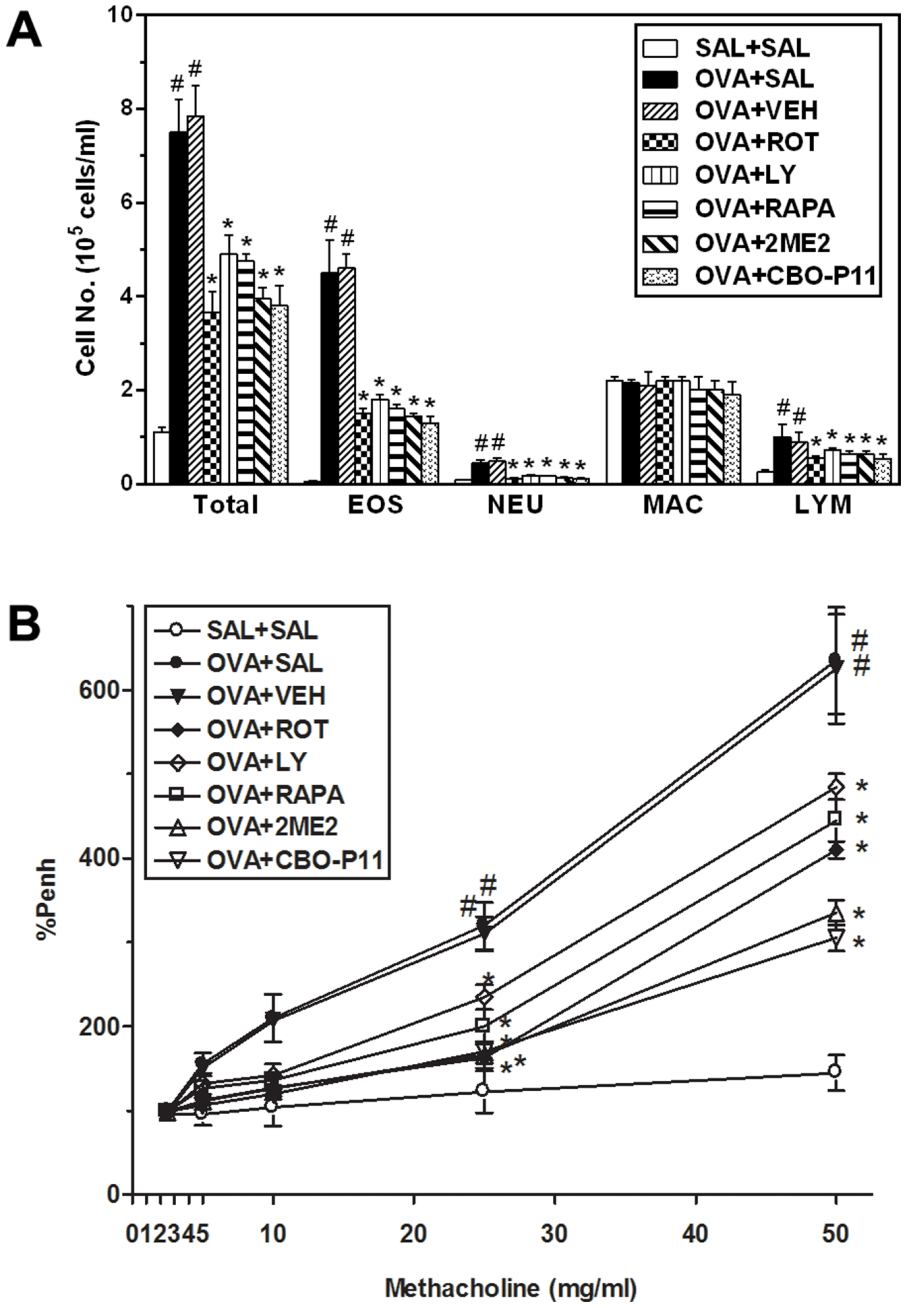
Effect of rottlerin, LY294002, rapamycin, 2ME2, or CBO-P11 on cellular changes in BAL fluids and airway responsiveness of OVA-inhaled mice. (A) The number of total and differential cellular components of BAL fluids. EOS (eosinophil), NEU (neutrophil), MAC (macrophage), and LYM (lymphocyte). (B) Airway hyperresponsiveness in response to methacholine. Results from four independent experiments with 5 mice/group are given as mean ± SEM. ^#^ *p*<0.05 vs SAL+SAL mice; *p<0.05 vs OVA+SAL mice. Sampling and whole body plethysmography was performed 48 h after the last challenge in mice. Saline-inhaled mice administered saline (SAL+SAL), OVA-inhaled mice administered saline (OVA+SAL), drug vehicle (OVA+VEH), rottlerin (OVA+ROT), LY294002 (OVA+LY), rapamycin (OVA+RAPA), 2-methoxyestradiol (OVA+2ME2), and CBO-P11 (OVA+CBO-P11).

During asthmatic attack, airway inflammation is marked by increased number of inflammatory cells in the airways and in the pulmonary subepithelial spaces [3]. We therefore, examined the effect of rottlerin (a PKC δ specific inhibitor), LY294002 (a PI3K inhibitor), or rapamycin (a mTOR inhibitor) on chemotaxis, that is, recruitment of inflammatory cells in BAL fluids after OVA challenge. As shown in Fig. 1A, numbers of total cells, eosinophils, neutrophils, and lymphocytes in BAL fluids is increased significantly at 48 h after OVA inhalation compared with the numbers after saline inhalation. The increased numbers of total cells, eosinophils, neutrophils, and lymphocytes are markedly reduced by the administration of rottlerin, LY294002, or rapamycin. In addition, 2ME2, an HIF-1α inhibitor or CBO-P11, a VEGF receptor inhibitor also blocks substantially the increase in cell counts after OVA inhalation.

### Effect of rottlerin, LY294002, rapamycin, 2ME2, or CBO-P11 on AHR of OVA-inhaled mice

AHR is indicative of chronic inflammation in the airways, which results from the airflow restriction due to mucus hypersecretion and remodeling leading to airway obstruction [34]. Airway responsiveness was assessed as a percentage of an increase in Penh in response to increasing doses of methacholine. In OVA-treated mice, the Penh dose-response curve is shifted to the left compared with the control curve (Fig. 1B). In addition, Penh produced by the administration of methacholine at 25 mg/ml is significantly greater in OVA-inhaled mice than in control mice. OVA-sensitized/challenged mice treated with rottlerin, LY294002, rapamycin, 2ME2, or CBO-P11 shows a substantial reduction in methacholine (25 mg/ml)-induced Penh compared with untreated mice after OVA inhalation. These results indicate that the administration of rottlerin, LY294002, rapamycin, 2ME2, or CBO-P11 reduces OVA-induced AHR in a murine model of asthma.

### Effect of rottlerin, LY294002, rapamycin, 2ME2, or CBO-P11 on lung inflammation of OVA-inhaled mice

**Figure 2:**
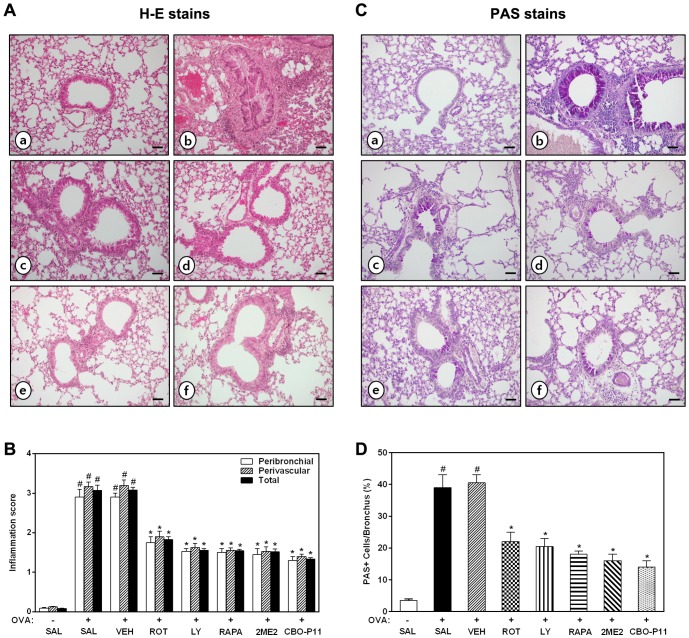
Effect of rottlerin, LY294002, rapamycin, 2ME2, or CBO-P11 on inflammatory cell recruitment and mucus hypersecretion in OVA-inhaled mice. (A, C) Representative of hematoxylin-eosin (H–E; A) and periodic acid-Schiff (PAS; C)-stained sections of the lungs. a, SAL+SAL; b, OVA+SAL; c, OVA+ROT; d, OVA+LY; e, OVA+RAPA; f, OVA+CBO-P11. Magnification ×200. Bars indicate 50 µm. (B) Inflammation scores. Total lung inflammation was defined as the average of the peribronchial and perivascular inflammation scores. (D) Quantitation of airway mucus expression. Sampling was performed 48 h after the last challenge in mice. Results from four independent experiments with 5 mice/group are given as mean ± SEM. ^#^ *p*<0.05 vs SAL+SAL; *p<0.05 vs OVA+SAL. Saline-inhaled mice administered saline (SAL+SAL), OVA-inhaled mice administered saline (OVA+SAL), drug vehicle (OVA+VEH), rottlerin (OVA+ROT), LY294002 (OVA+LY), rapamycin (OVA+RAPA), 2-methoxyestradiol (OVA+2ME2), and CBO-P11 (OVA+CBO-P11).

The above-mentioned reduction in chemotaxis into the airway correlates with the histological changes of lung parenchyma. Lungs from OVA-sensitized/challenged mice show widespread peribronchiolar and perivascular inflammatory cell infiltrates compared with those from saline-inhaled mice (Fig. 2A and B). However, the administration of rottlerin, LY294002, rapamycin, 2ME2, or CBO-P11 results in a significant reduction of inflammatory cell infiltration. These results indicate that treatment with rottlerin, LY294002, rapamycin, 2ME2, or CBO-P11 efficiently inhibits the infiltration of inflammatory cells and attenuates allergic airway inflammation.

### Effect of rottlerin, LY294002, rapamycin, 2ME2, or CBO-P11 on airway mucus expression in lungs of OVA-inhaled mice

**Figure 3:**
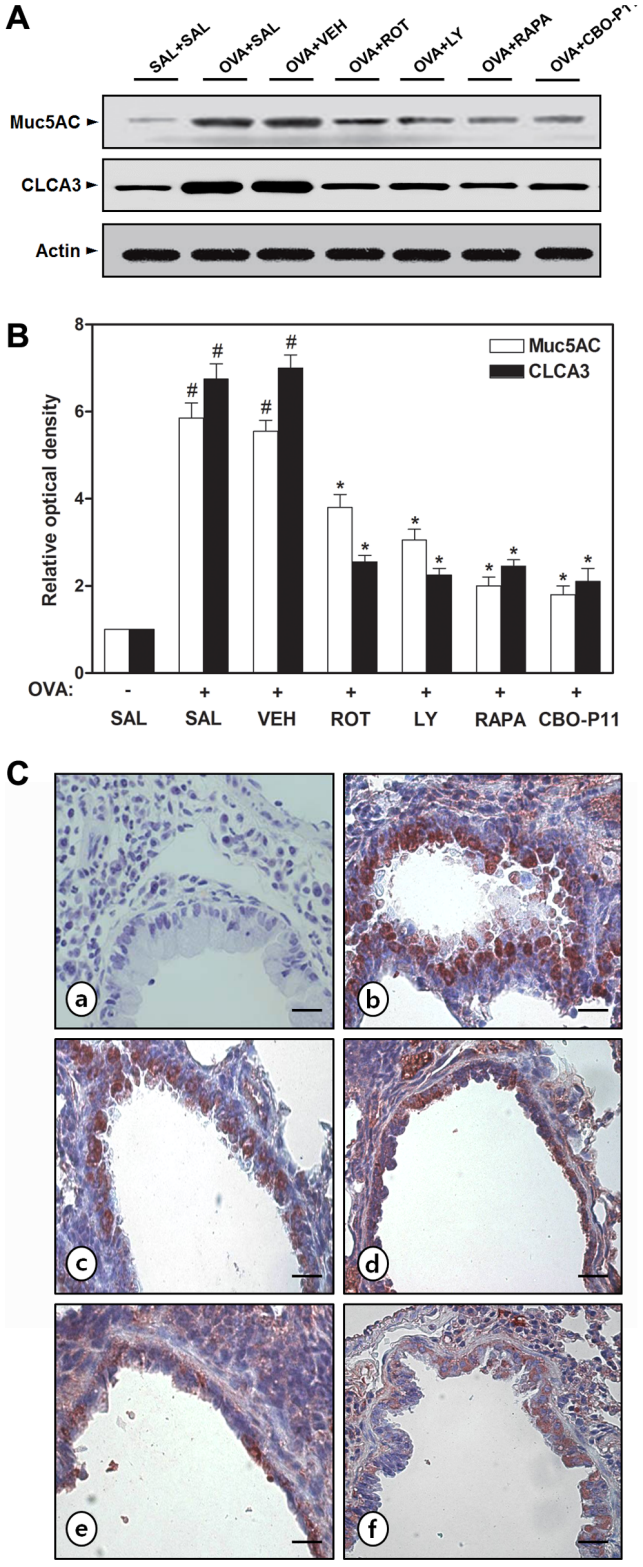
Effect of rottlerin, LY294002, rapamycin, or CBO-P11 on the protein expression of Muc5AC and CLCA3 in OVA-inhaled mice. (A) Western blotting of Muc5AC and CLCA3 in lung tissues. (B) Densitometric analyses are presented as the relative ratio of each molecule to actin. The relative ratio of each molecule in the lung tissues of SAL+SAL mice is arbitrarily presented as 1. (C) Localization of immunoreactive Muc5AC in the bronchiolar epithelial layer. a, SAL+SAL; b, OVA+SAL; c, OVA+ROT; d, OVA+LY; e, OVA+RAPA; f, OVA+CBO-P11. Magnification ×200. Bars indicate 50 µm. Sampling was performed 48 h after the last challenge in mice. Results from four independent experiments with 5 mice/group are given as mean ± SEM. ^#^ *p*<0.05 vs SAL+SAL; *p<0.05 vs OVA+SAL. Saline-inhaled mice administered saline (SAL+SAL), OVA-inhaled mice administered saline (OVA+SAL), drug vehicle (OVA+VEH), rottlerin (OVA+ROT), LY294002 (OVA+LY), rapamycin (OVA+RAPA), and CBO-P11 (OVA+CBO-P11).

Since AHR has direct correlation with the excessive mucus secretion that is responsible for airway obstruction [34], we determined the effect of rottlerin, LY294002, rapamycin, 2ME2, or CBO-P11 on mucus hypersecretion in an OVA-induced murine model of asthma. The mice lung sections were stained with PAS and the number of PAS-positive cells was determined. The percentage of epithelial goblet cells that is stained positively with PAS in OVA-inhaled mice is substantially greater than that in saline-inhaled mice (Fig 2C and D). The increased levels of PAS-positive airway epithelium after OVA inhalation are decreased significantly by the treatment of rottlerin, LY294002, rapamycin, 2ME2, or CBO-P11. To further assess the goblet cell response, the protein levels of Muc5AC and CLCA3 were measured by Western blot analysis. The expression of Muc5AC and CLCA3 is markedly increased in OVA-inhaled mice compared with saline-inhaled mice. Rottlerin, LY294002, rapamycin, or CBO-P11 significantly reduces the protein expression of Muc5AC and CLCA3 (Fig. 3A and B). Abundant goblet cells are also detected in the airways of asthmatic mice by Muc5AC immunostaining, whereas no staining is detectable in saline-inhaled controls (Fig. 3C). Muc5AC staining for goblet cells is remarkably attenuated by the treatment of rottlerin, LY294002, rapamycin, or CBO-P11.

### Effect of rottlerin, LY294002, or rapamycin on release of total and OVA-specific IgE into BAL fluids of OVA-inhaled mice

**Figure 4:**
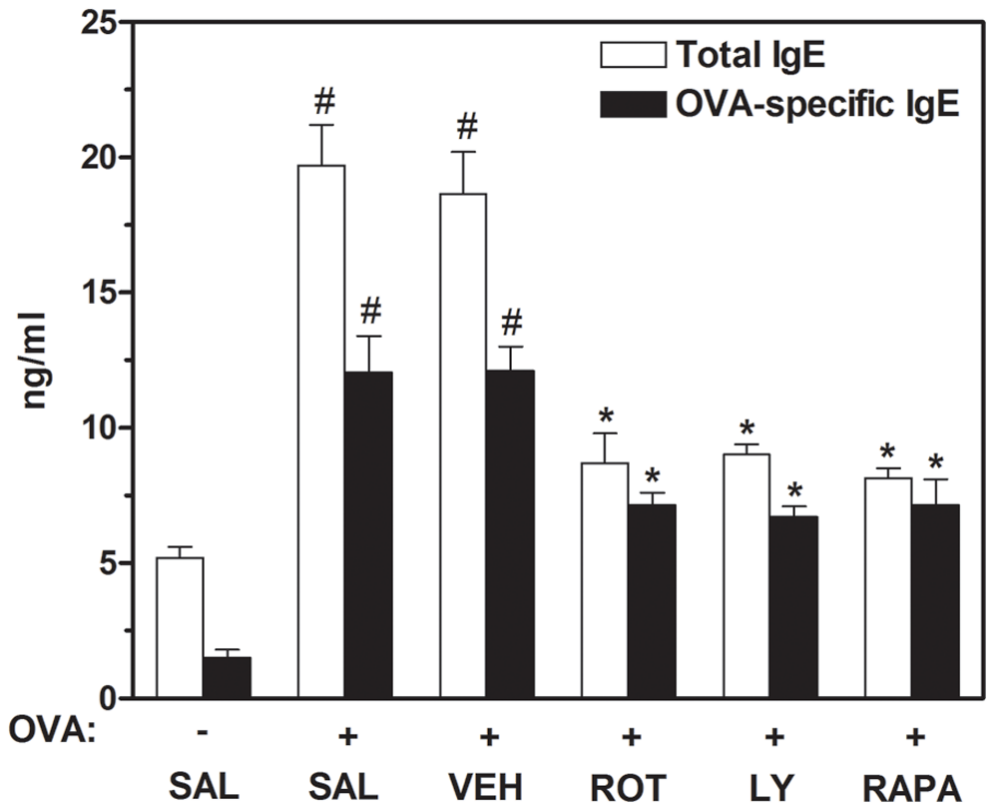
Effect of rottlerin, LY294002, or rapamycin on total and OVA-specific IgE levels in BAL fluids of OVA-inhaled mice. The levels of total and OVA-specific IgE were quantified by ELISA. Results from four independent experiments with 5 mice/group are given as mean ± SEM. ^#^ *p*<0.05 vs SAL+SAL; *p<0.05 vs OVA+SAL. Saline-inhaled mice administered saline (SAL+SAL), OVA-inhaled mice administered saline (OVA+SAL), drug vehicle (OVA+VEH), rottlerin (OVA+ROT), LY294002 (OVA+LY), and rapamycin (OVA+RAPA).

Total and OVA-specific IgE levels were determined by ELISA in each experimental group. IgE levels in BAL fluids are dramatically elevated in OVA-challenged mice, compared with control mice. However, the administration of rottlerin, LY294002, or rapamycin to OVA-inhaled mice leads to a significant reduction in the total and OVA-specific IgE levels (Fig. 4).

### Effect of rottlerin, LY294002, or rapamycin on the levels of Th2 cytokines and proinflammatory cytokines in OVA-inhaled mice

**Figure 5:**
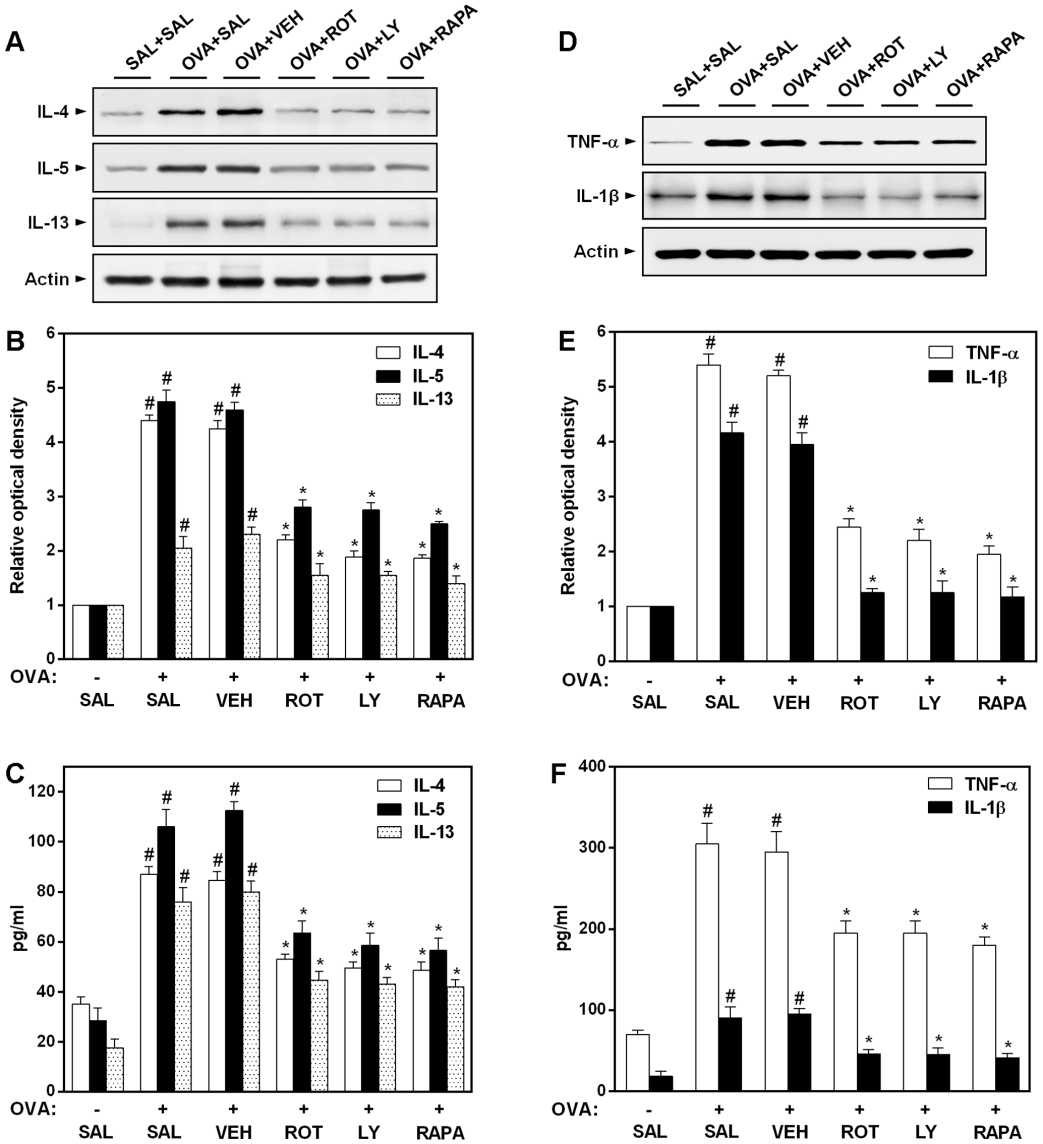
Effect of rottlerin, LY294002, or rapamycin on Th2 and proinflammatory cytokine levels in OVA-inhaled mice. (A, D) Western blotting of IL-4, IL-5, IL-13, TNF-α, and IL-1β in lung tissues. (B, E) Densitometric analyses are presented as the relative ratio of each molecule to actin. The relative ratio of each molecule in the lung tissues of SAL+SAL mice is arbitrarily presented as 1. (C, F) The levels of IL-4, IL-5, IL-13, TNF-α, and IL-1β were quantified by ELISA. The protein expression of these cytokines was measured at 48 h after the last challenge in mice. Results from four independent experiments with 5 mice/group are given as mean ± SEM. ^#^ *p*<0.05 vs SAL+SAL; *p<0.05 vs OVA+SAL. Saline-inhaled mice administered saline (SAL+SAL), OVA-inhaled mice administered saline (OVA+SAL), drug vehicle (OVA+VEH), rottlerin (OVA+ROT), LY294002 (OVA+LY), and rapamycin (OVA+RAPA).

Allergic asthmatic inflammation is known to be caused by the secretion of a series of Th2 cytokines (IL-4, IL-5, and IL-13) and proinflammatory cytokines (TNF-α and IL-1β) [1]. To assess the effect of rottlerin, LY294002, or rapamycin on pulmonary inflammation in allergic mice, levels of these cytokines in BAL fluids as well as lung tissues were measured. Western blot analysis reveals that protein expression of IL-4, IL-5, IL-13, TNF-α, and IL-1β in lung tissues is significantly upregulated in OVA-inhaled mice compared with that in saline-inhaled mice (Fig. 5A, B, D, and E). The elevated levels of these cytokines after OVA challenge are significantly reduced by rottlerin, LY294002, or rapamycin. Consistent with these results, ELISA shows that the levels of IL-4, IL-5, IL-13, TNF-α, and IL-1β in BAL fluids are significantly increased in OVA-challenged mice compared with the levels in control mice (Fig. 5C and F). The increased levels of these cytokines are significantly decreased by the administration of rottlerin, LY294002, or rapamycin. Meanwhile, 2ME2 or CBO-P11 also efficiently suppresses the increment of Th2 and proinflammatory cytokine levels in BAL fluids of OVA-inhaled mice (data not shown).

### Effect of rottlerin, LY294002, or rapamycin on eotaxin, ICAM-1, and VCAM-1 expression in lungs of OVA-inhaled mice

**Figure 6:**
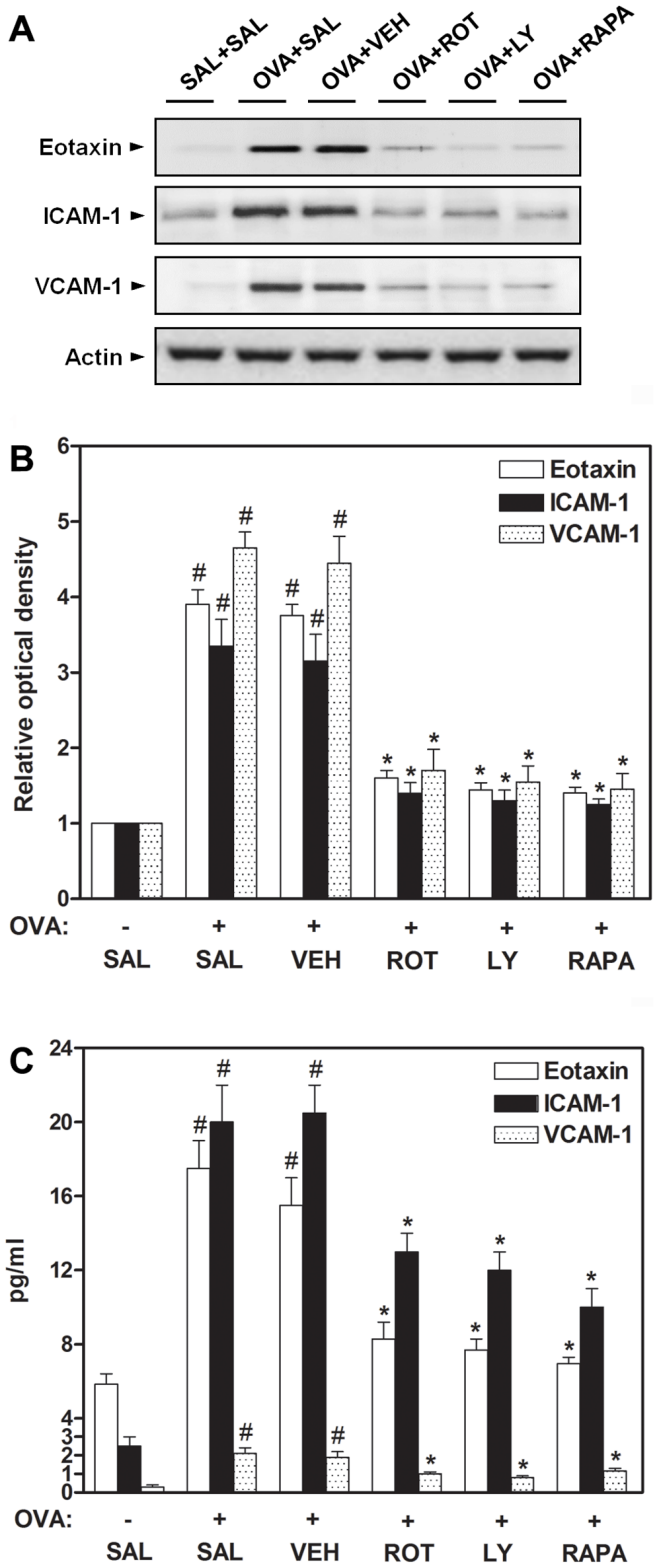
Effect of rottlerin, LY294002, or rapamycin on eotaxin, ICAM-1, and VCAM-1 levels in OVA-inhaled mice. (A) Western blotting of eotaxin, ICAM-1, and VCAM-1 in lung tissues. (B) Densitometric analyses are presented as the relative ratio of each molecule to actin. The relative ratio of each molecule in the lung tissues of SAL+SAL mice is arbitrarily presented as 1. (C) The levels of eotaxin, ICAM-1, and VCAM-1 were quantified by ELISA. The protein expression of these adhesion molecules was measured at 48 h after the last challenge in mice. Results from four independent experiments with 5 mice/group are given as mean ± SEM. ^#^ *p*<0.05 vs SAL+SAL; *p<0.05 vs OVA+SAL. Saline-inhaled mice administered saline (SAL+SAL), OVA-inhaled mice administered saline (OVA+SAL), drug vehicle (OVA+VEH), rottlerin (OVA+ROT), LY294002 (OVA+LY), and rapamycin (OVA+RAPA).

Leukocyte-endothelial adhesion molecules (ICAM-1 and VCAM-1) and chemokines (eotaxin) are important in the recruitment and migration of leukocytes to the sites of inflammation [35], [36]. Expression of these molecules is modulated by cytokines such as TNF-α, IL-1β, and IL-4 [37]. In view of the suppressive effect of rottlerin, LY294002, or rapamycin on these cytokine levels, their effect on eotaxin, ICAM-1, and VCAM-1 expression in OVA-treated mice was examined. Western blot analysis reveals that the protein levels of eotaxin, ICAM-1, and VCAM-1 in lung tissues are increased at 48 h after OVA inhalation compared with the levels after saline inhalation. The increased levels of these proteins are reduced by the administration of rottlerin, LY294002, or rapamycin (Fig. 6A and B). Similarly, ELISA shows that the levels of eotaxin, ICAM-1, and VCAM-1 in BAL fluids are significantly increased in OVA-challenged mice compared with the levels in control mice (Fig. 6C). The increased levels of these molecules are significantly decreased by the administration of rottlerin, LY294002, or rapamycin. Likewise, 2ME2 or CBO-P11 efficiently represses the increment in the expressions of adhesion molecules and chemokine in BAL fluids of OVA-inhaled mice (data not shown).

### Effect of rottlerin, LY294002, rapamycin, 2ME2, or CBO-P11 on plasma extravasation in OVA-inhaled mice

**Figure 7:**
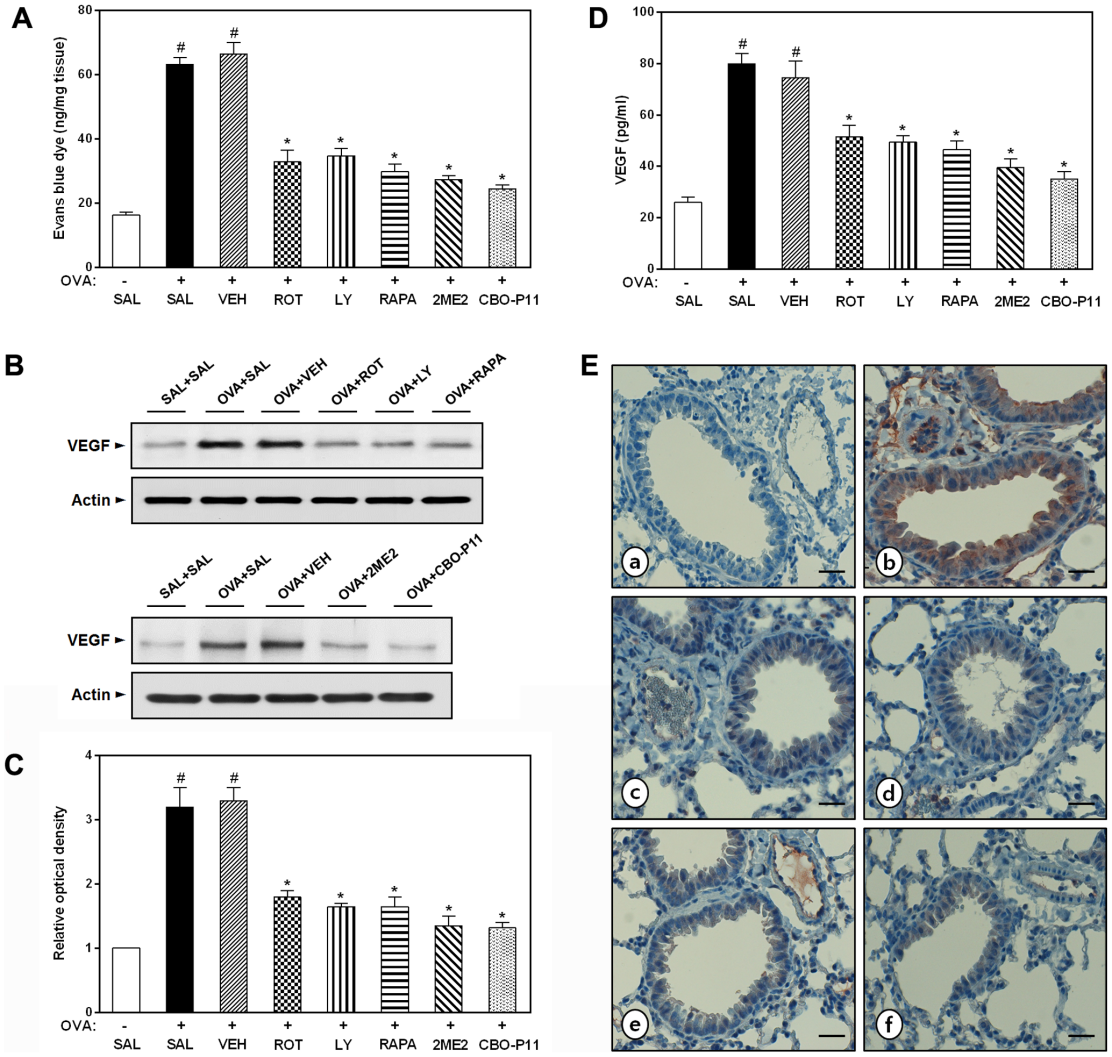
Effect of rottlerin, LY294002, rapamycin, 2ME2, or CBO-P11 on plasma extravasation and VEGF expression in OVA-inhaled mice. (A) Evans blue dye assay of plasma extravasation. (B) Western blotting of the levels of VEGF in lung tissues. (C) Densitometric analyses are presented as the relative ratio of each molecule to actin. The relative ratio of each molecule in the lung tissues of SAL+SAL mice is arbitrarily presented as 1. (D) The levels of VEGF were quantified by ELISA. (E) Localization of immunoreactive VEGF in the bronchiolar epithelial layer. a, SAL+SAL; b, OVA+SAL; c, OVA+ROT; d, OVA+LY; e, OVA+RAPA; f, OVA+CBO-P11. Magnification ×200. Bars indicate 50 µm. The protein levels of VEGF were measured at 48 h after the last challenge in mice. Results from four independent experiments with 5 mice/group are given as mean ± SEM. ^#^ *p*<0.05 vs SAL+SAL; *p<0.05 vs OVA+SAL. Saline-inhaled mice administered saline (SAL+SAL), OVA-inhaled mice administered saline (OVA+SAL), drug vehicle (OVA+VEH), rottlerin (OVA+ROT), LY294002 (OVA+LY), rapamycin (OVA+RAPA), 2-methoxyestradiol (OVA+2ME2), and CBO-P11 (OVA+CBO-P11).

EBD assay reveals that plasma extravasation is significantly increased at 48 h after the last challenge of OVA (Fig. 7A). The increase of plasma extravasation after OVA inhalation is significantly reduced by the administration of rottlerin, LY294002, rapamycin, 2ME2, or CBO-P11.

### Effect of rottlerin, LY294002, rapamycin, 2ME2, or CBO-P11 on VEGF protein levels in OVA-inhaled mice

Western blot analysis reveals that the increased VEGF levels at 48 h after OVA inhalation are decreased significantly by the administration of rottlerin, LY294002, rapamycin, 2ME2, or CBO-P11 (Fig. 7B and C). Consistent with these results, ELISA shows that the increased VEGF levels in BAL fluids after OVA inhalation are decreased markedly by the administration of rottlerin, LY294002, rapamycin, 2ME2, or CBO-P11 (Fig. 7D). Further, immunohistochemical analyses show the localization of immunoreactive VEGF on inflammatory cells, endothelium of pulmonary vessels, and the extracellular matrix around each bronchus in OVA-induced asthmatic lungs (Fig. 7E). However, immunoreactive VEGF is substantially reduced in lung tissues from saline-inhaled mice treated with saline and OVA-inhaled mice treated with rottlerin, LY294002, rapamycin, or CBO-P11.

### Effect of rottlerin, LY294002, rapamycin, or 2ME2, on HIF-1α protein levels in OVA-inhaled mice

**Figure 8:**
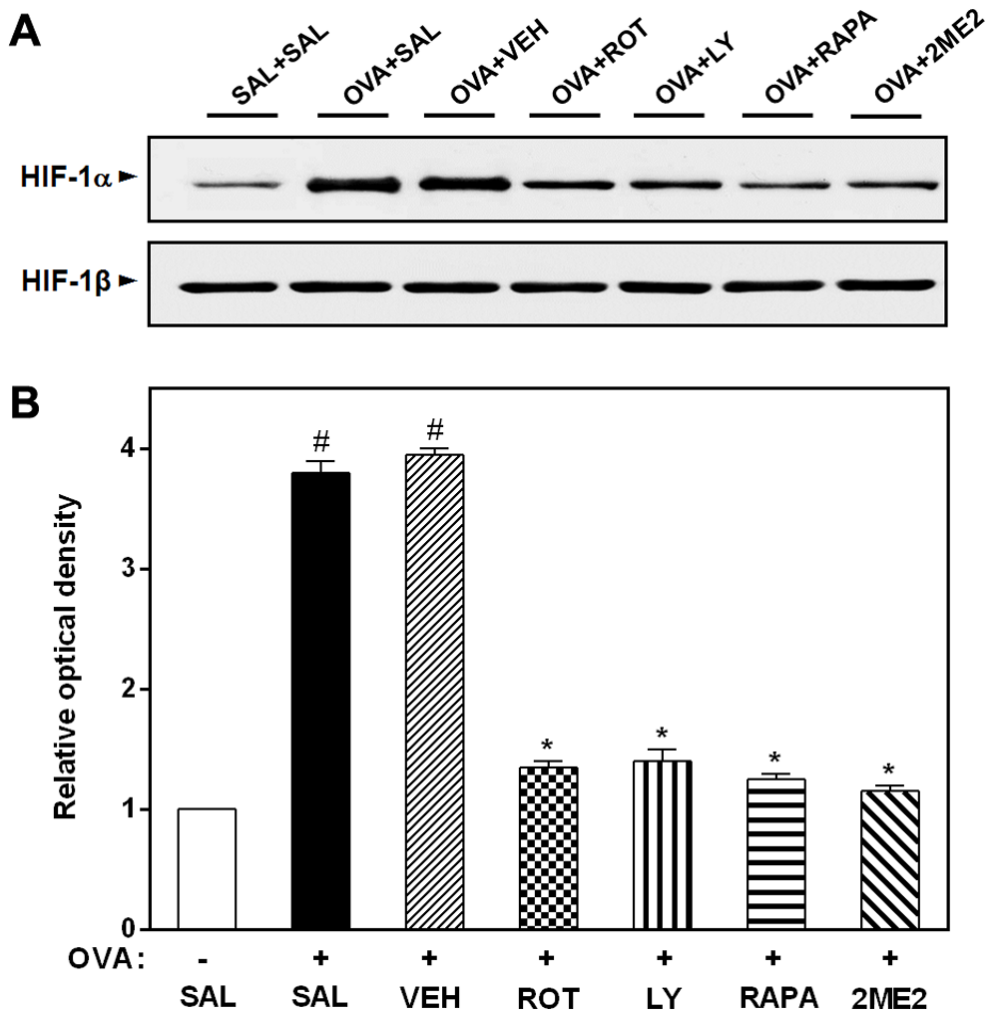
Effect of rottlerin, LY294002, rapamycin, or 2ME2 on HIF-1α levels in nuclear protein extracts from lung tissues of OVA-inhaled mice. (A) Western blotting of the levels of HIF-1α in lung tissues. (B) Densitometric analyses are presented as the relative ratio of each molecule to actin. The relative ratio of each molecule in the lung tissues of SAL+SAL mice is arbitrarily presented as 1. The protein levels of HIF-1α were measured at 48 h after the last challenge in mice. Results from four independent experiments with 5 mice/group are given as mean ± SEM. ^#^ *p*<0.05 vs SAL+SAL; *p<0.05 vs OVA+SAL. Saline-inhaled mice administered saline (SAL+SAL), OVA-inhaled mice administered saline (OVA+SAL), drug vehicle (OVA+VEH), rottlerin (OVA+ROT), LY294002 (OVA+LY), rapamycin (OVA+RAPA), and 2-methoxyestradiol (OVA+2ME2).

To investigate whether the activation of HIF-1α is regulated by PKC δ, PI3K/Akt, or mTOR in allergic airway disease, we administered rottlerin, LY294002, rapamycin, or 2ME2, *in vivo*, measuring the HIF-1α activity in the lung tissues from OVA-inhaled mice. Western blot analysis shows that the administration of rottlerin, LY294002, rapamycin, or 2ME2 reduces substantially the increased HIF-1α levels in nuclear protein extracts of lung tissues from OVA-inhaled mice (Fig. 8).

### Effect of rottlerin, LY294002, or rapamycin on phosphorylation of mTOR and p70S6K in OVA-inhaled mice

**Figure 9:**
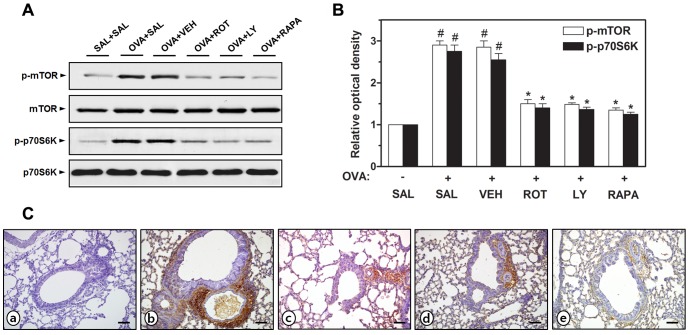
Effect of rottlerin, LY294002, or rapamycin on phosphorylation of mTOR and p70S6K in OVA-inhaled mice. (A) Western blotting of the levels of phosphorylated (p-) mTOR, mTOR, p-p70S6K, and p70S6K in lung tissues. (B) Densitometric analyses are presented as the relative ratio of each molecule to actin. The relative ratio of each molecule in the lung tissues of SAL+SAL mice is arbitrarily presented as 1. (C) Localization of immunoreactive p-mTOR in the bronchiolar epithelial layer. a, SAL+SAL; b, OVA+SAL; c, OVA+ROT; d, OVA+LY; e, OVA+RAPA. Magnification ×200. Bars indicate 50 µm. The levels of these proteins were measured at 48 h after the last challenge in mice. Results from four independent experiments with 5 mice/group are given as mean ± SEM. ^#^ *p*<0.05 vs SAL+SAL; *p<0.05 vs OVA+SAL. Saline-inhaled mice administered saline (SAL+SAL), OVA-inhaled mice administered saline (OVA+SAL), drug vehicle (OVA+VEH), rottlerin (OVA+ROT), LY294002 (OVA+LY), and rapamycin (OVA+RAPA).

Considering the established relationship among PKC δ, PI3K/Akt, and mTOR in various cellular conditions [25], [38], we hypothesized that PKC δ and PI3K/Akt might be upstream molecules of mTOR signaling pathway in allergic airway disease. Therefore, we examined whether the administration of a PKC δ inhibitor or a PI3K inhibitor could attenuate the phosphorylation of mTOR and its effector p70S6K in OVA-treated mice. Western blot analysis reveals that the administration of rottlerin, LY294002, or rapamycin, prevents the phosphorylation of mTOR and p70S6K in lung tissues from OVA-inhaled mice (Fig. 9A and B). Immunohistochemical analyses also demonstrate that strong phosphorylated (p-) mTOR immunoreactivity is present in the lung epithelium of OVA-inhaled mice, while the lungs of control mice show minimal staining (Fig. 9C). The administration of rottlerin, LY294002, or rapamycin represents a significantly decreased immunoreactivity of p-mTOR in OVA-challenged lungs. From these, it is inferred that PKC δ and PI3K/Akt might activate mTOR and p70S6K in an experimental asthma model.

### Effect of rottlerin or LY294002 on phosphorylation of PKC δ, PI3K, and Akt in lungs of OVA-inhaled mice

**Figure 10:**
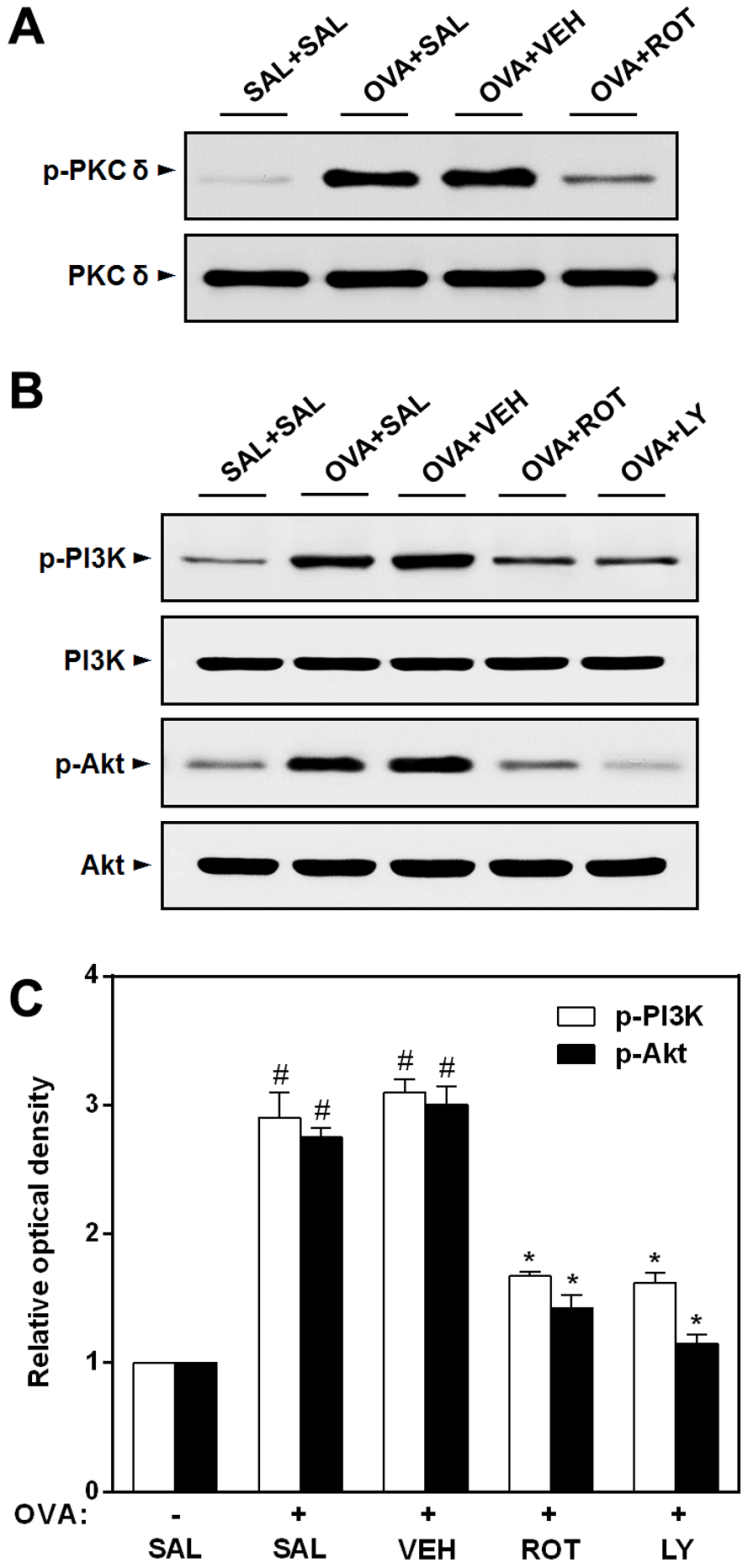
Effect of rottlerin or LY294002 on phosphorylation of PKC δ, PI3K, and Akt in lung tissues of OVA-inhaled mice. (A) Western blotting of the levels of phosphorylated (p-) PKC δ, PKC δ in lung tissues. (B) Western blotting of the levels of p-PI3K, PI3K, p-Akt, and Akt in lung tissues. (C) Densitometric analyses are presented as the relative ratio of each molecule to actin. The relative ratio of each molecule in the lung tissues of SAL+SAL mice is arbitrarily presented as 1. The levels of these proteins were measured at 48 h after the last challenge in mice. Results from four independent experiments with 5 mice/group are given as mean ± SEM. ^#^ *p*<0.05 vs SAL+SAL; *p<0.05 vs OVA+SAL. Saline-inhaled mice administered saline (SAL+SAL), OVA-inhaled mice administered saline (OVA+SAL), drug vehicle (OVA+VEH), rottlerin (OVA+ROT), and LY294002 (OVA+LY).

Collectively, the results presented above strongly suggest that PKC δ and PI3K/Akt are specially required not only for mTOR phosphorylation but also for subsequent HIF-1α-mediated VEGF expression in OVA-treated mice. Next, to determine whether PKC δ is a upstream mediator of PI3K/Akt signaling, our group tested the effect of a PKC δ specific inhibitor, rottlerin on the phosphorylation of PKC δ, PI3K, and Akt in OVA-inhaled mice. As shown in Fig. 10, levels of p-PKC δ, p-PI3K, and p-Akt (Ser^473^) in the lung tissues are significantly increased at 48 h after OVA inhalation compared with levels seen in control mice. However, no significant changes in the levels of PKC δ, PI3K, and Akt are observed in any of the groups tested. The increased levels of p-PKC δ, p-PI3K, and p-Akt in the lung tissues after OVA inhalation are markedly reduced by the administration of rottlerin, suggesting that PKC δ might act upstream of PI3K/Akt pathway in a murine model of asthma.

### Time course study of protein levels of p-PKC δ, p-Akt, p-mTOR, HIF-1α, and VEGF in lungs from OVA-inhaled mice

**Figure 11:**
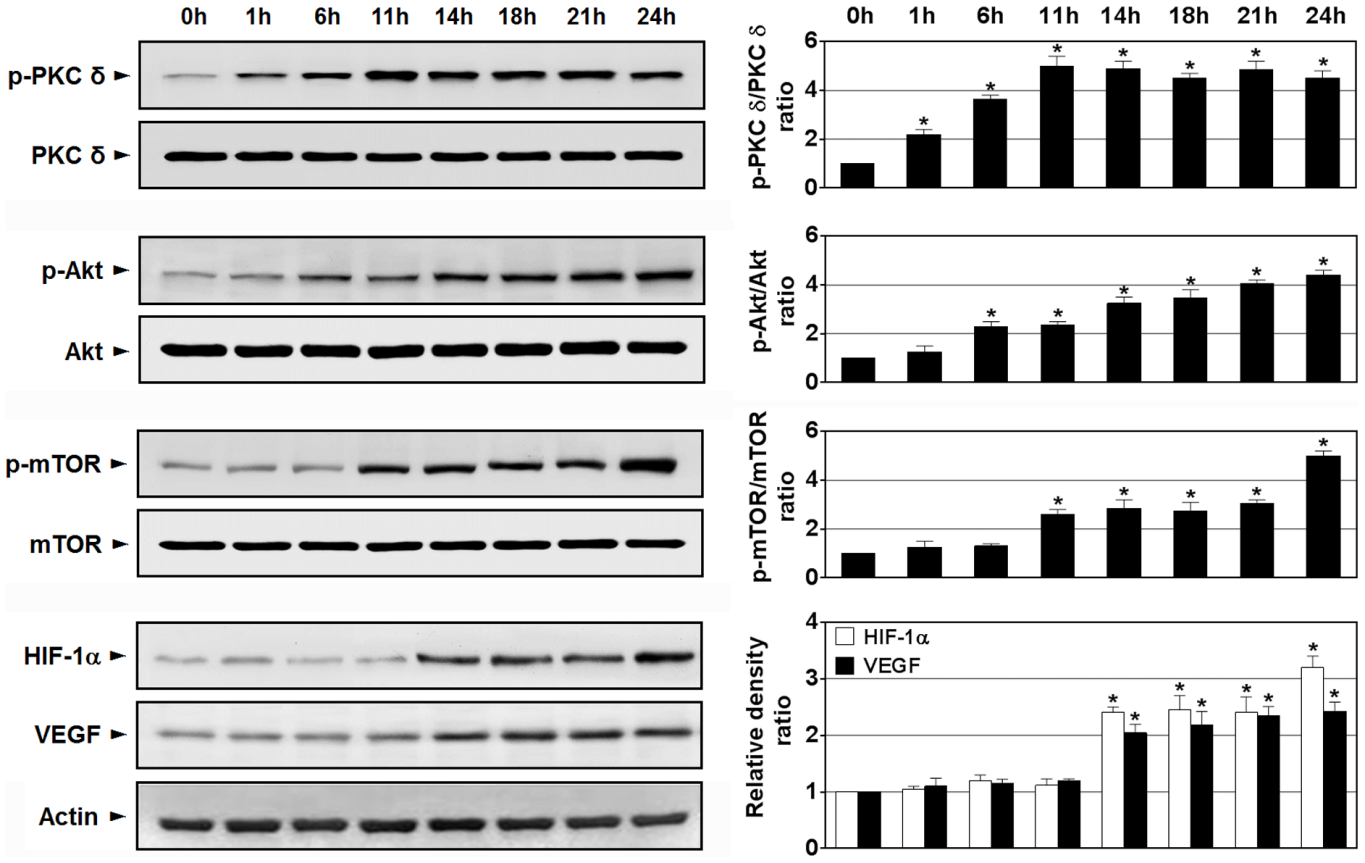
Time course analysis of phosphorylation of PKC δ and other signaling intermediates in lung tissues of OVA-inhaled mice. Western blotting of the levels of phosphorylated (p-) PKC δ, PKC δ, p-Akt, Akt, p-mTOR, mTOR, HIF-1α, and VEGF in lung tissues. Representative images with respective densitometry data (bar diagram) are shown. Results from four independent experiments with 5 mice/group are given as mean ± SEM. *p<0.05 vs the prechallenge group (0 h).

As shown in Fig. 11, Western blot analysis reveals that the protein levels of p-PKC δ, p-Akt, p-mTOR, HIF-1α, and VEGF start to increase significantly at 1, 6, 11, 14 and 14 h, respectively, after challenge with OVA compared with levels seen in the prechallenge period (0 h). In contrast, no significant changes in levels of PKC δ, Akt, and mTOR are observed in OVA-challenged lungs.

### Effect of rottlerin or siRNA-PKC δ on TNF-α/IL-4-induced phosphorylation of PKC δ, Akt, mTOR, p70S6K, HIF-1α, and VEGF in BEAS-2B cells

**Figure 12:**
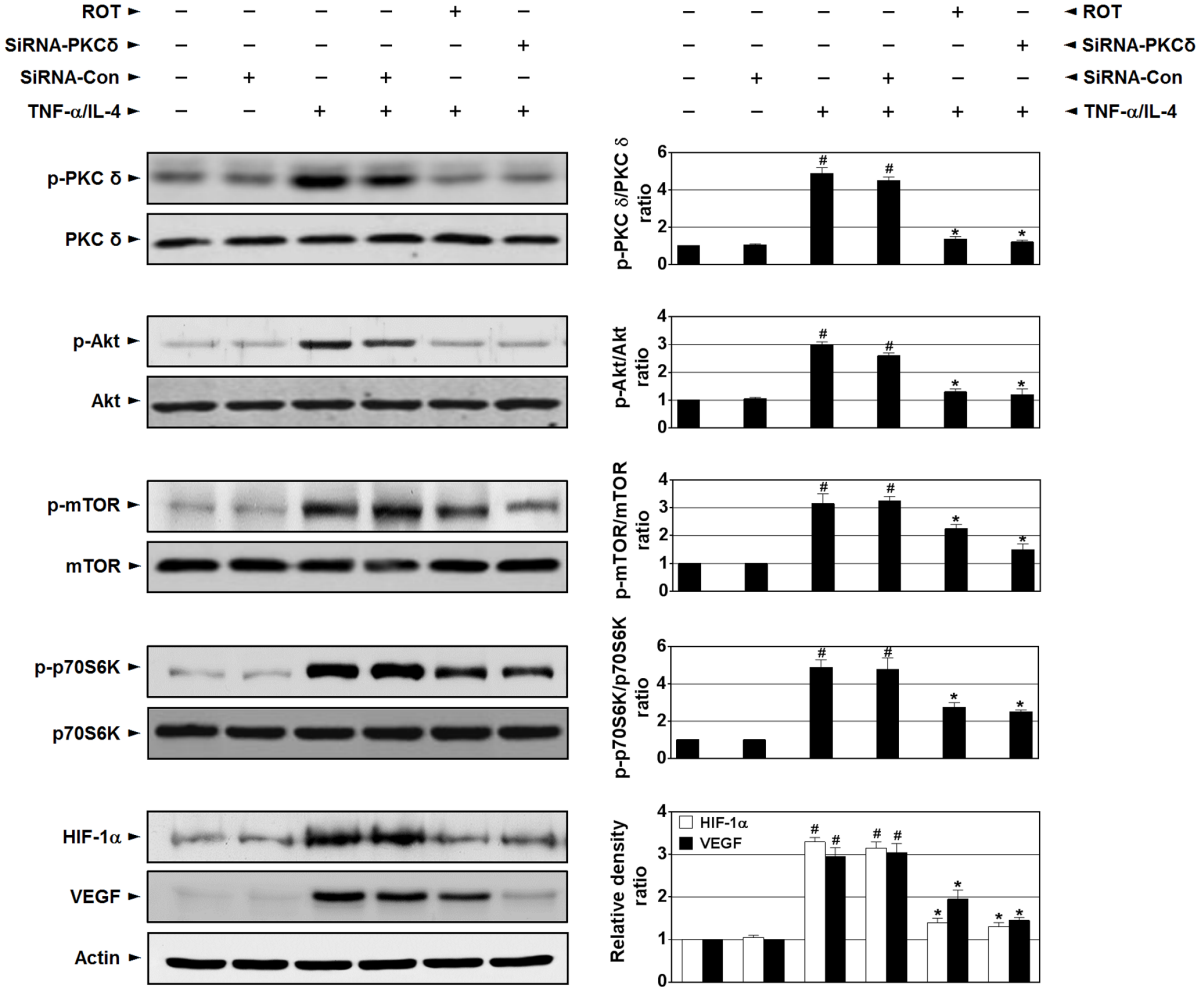
Effect of rottlerin or siRNA-PKC δ on phosphorylation of PKC δ and other signaling intermediates in TNF-α/IL-4-treated BEAS-2B cells. Western blotting of the levels of phosphorylated (p-) PKC δ, PKC δ, and other signaling intermediates in TNF-α/IL-4-treated BEAS-2B cells. BEAS-2B cells were pretreated with rottlerin (ROT, 4 µM) for 6 h prior to challenge with TNF-α/IL-4 for the indicated time periods or transfected with siRNA targeting PKC δ (siRNA-PKC δ) or control siRNA (siRNA-Con) and then challenged with TNF-α/IL-4. Representative images with respective densitometry data (bar diagram) are shown. Results from four independent experiments with 5 mice/group are given as mean ± SEM. ^#^ *p*<0.05 vs the untreated group; *p<0.05 vs the group treated by TNF-α/IL-4.

To further confirm that PKC δ is closely involved in PI3K/Akt/mTOR/HIF-1α/VEGF pathway, Western blot analyses of the expression of p-PKC δ, p-Akt, p-mTOR, p-p70S6K, HIF-1α, and VEGF in BEAS-2B cells were performed. According to our results, the levels of p-PKC δ, p-Akt, p-mTOR, p-p70S6K, HIF-1α, and VEGF are increased at 24 h in BEAS-2B cells treated by TNF-α/IL-4 (Fig. 12). The elevated levels of these proteins are significantly decreased by rottlerin or siRNA-PKC δ.

## Discussion

Asthmatic airway inflammation is usually accompanied by increased vascular permeability and plasma extravasation [1]. Although several inflammatory mediators, including platelet-activating factor and nitric oxide, could promote microvascular leakage [39], it is apparent that VEGF is a major determinant of vascular permeability enhancement [40]. During an asthma attack, VEGF allows plasma proteins to leak into extravascular spaces, which induces a thickened, engorged, and edematous airway wall, resulting in narrowing of the airway lumen and profound alterations of the endothelial cell matrix [40]. VEGF also stimulates leukocyte migration to endothelial cells by promoting ICAM-1 and VCAM-1 expression, suggesting the proinflammatory role of VEGF in allergic response [41]. Furthermore, overexpression of VEGF induces enhanced allergic sensitization, upregulation of Th2-type inflammatory responses, and mucous gland hyperplasia [42]. Thus, inhibition of VEGF activity has been proposed as a potential therapeutic strategy in allergic airway disease. In agreement with these findings, we have shown that OVA-exposed mice have the increased levels of VEGF, IgE, Th2 cytokines (IL-4, IL-5, and IL-13), proinflammatory cytokines (TNF-α and IL-1β), eotaxin, and adhesion molecules (ICAM-1 and VCAM-1), which parallels with the development of typical asthmatic features such as eosinophilic inflammation, AHR, goblet cell metaplasia, and increased vascular permeability. However, the augmentation in these key mediators and allergic symptoms is significantly reduced by the administration of a PKC δ inhibitor, rottlerin, or a VEGF inhibitor, CBO-P11. Therefore, it is proposed that PKC δ exerts an important role in inducing and maintaining Th2-mediated allergic airway inflammation by upregulating VEGF expression.

HIF-1α is known to be one of the key factors regulating inflammatory responses [22]. Thus, there have been some close links between unfavorable clinical outcome and HIF-1α overexpression [43], [44]. As well in studies on asthma, it has been found that overexpression of HIF-1α is involved in the development of allergic airway inflammation [45]. In the present study, the determination of HIF-1α protein levels in nuclear extracts has revealed that these protein levels are substantially increased in OVA-induced asthmatic mice, suggesting that HIF-1α is activated. The increased levels of HIF-1α are significantly reduced after the administration of rottlerin. These findings, therefore, implies that PKC δ might upregulate HIF-1α activity in allergic airway disease. As mentioned above, HIF-1α modulates VEGF transcription by binding to the VEGF promoter [22]. Recent evidence also indicates that the increased expression of VEGF is decreased by the inhibition of HIF-1α activity in OVA-inhaled mice [26]. Consistent with these, our study has shown that 2ME2, a HIF-1α inhibitor, attenuates the OVA-induced increase of VEGF levels and ameliorates typical asthmatic symptoms in allergic mice, as does rottlerin. Taken together, PKC δ inhibition with rottlerin is suggested to reduce VEGF expression through the downregulation of HIF-1α activity in OVA-challenged mice. Indeed, PKC δ appears to be an important component for regulating the HIF-1α-VEGF module.

mTOR has been drawing considerable attention because it stands at the intersection of an important multiple signaling pathway [14]. Activated mTOR phosphorylates at least two targets, p70S6K and 4E-BP1, which in turn activates p70S6K and inhibits 4E-BP1. These two components lead to active translation of mRNAs in which HIF-1α expression is involved [46]. Generally, mTOR activation is known to be necessary for the differentiation and proliferation of CD4^+^ effector T cells into Th2 and Th17 subsets [47]. Supporting this contention, the mTOR inhibitor rapamycin has been reported to block airway inflammation in HDM-sensitized mice, which is mediated by the reduced expression of Th2- and Th17-type cytokines [17]. mTOR inhibition also has effects on other immune cells that participate in the pathogenesis of asthma [48]. Rapamycin suppresses B cell responses and antibody production, attenuates neutrophil chemotaxis, and prevents natural killer T cell proliferation. On the other hand, cumulative evidence suggests that mTOR pathway is vital for airway and vascular remodeling, which is a key characteristic of severe asthma. Above all, mTOR signaling appears to be implicated in the airway smooth muscle hyperplasia and hypertrophy seen in chronic asthma [49], [50]. Rapamycin prevents airway myocyte differentiation into a contractile phenotype via blockade of the mTOR-p70S6K pathway [51]. It also inhibits transforming growth factor-α-induced pulmonary fibrotic response, which could contribute to subepithelial fibrosis and airway remodeling [52]. Further, mTOR may regulate angiogenesis and lymphangiogenesis, both of which play crucial roles in pulmonary vascular remodeling [53], [54].

Although the above findings highlight the importance of the mTOR pathway in allergic asthma, studies in OVA or HDM models did not describe the downstream cascade of mTOR pathway [17]–[20]. Thus, we have investigated whether rapamycin could provide new insights into addressing this question. In the present study, rapamycin blocks OVA-induced increases in bronchial inflammation, AHR, levels of Th2 and proinflammatory cytokines and adhesion molecules, and vascular permeability, suggesting that rapamycin can inhibit cardinal features of allergic asthma. These results seem to be similar to other reports, which describe the effects of rapamycin or its derivative (SAR 943) on experimental asthma models [17]–[20]. However, regarding the impact of these reagents on AHR, IgE production, or Th2 cytokine profile, there is a partial discrepancy between our study and prior data. For example, Nagai *et al* has shown that mice sensitized with OVA and treated with rapamycin reveal reductions in IgE levels; however, no change in eosinophils or AHR is observed [18]. In contrast, a similar study in mice has demonstrated attenuation of eosinophils, IL-4, goblet cells, and AHR responses after SAR 943 treatment but no effect on serum IgE levels [19]. The exact reason for these contradictory results is not clear, but it may be due to variations in the experimental conditions, such as mice strains, sensitization and challenge protocols, doses, or assessment of AHR. Meanwhile, we have shown that OVA-exposed mice have the marked increase in levels of p-mTOR and p-p70S6K compared with saline controls, confirming the activation of mTOR by OVA challenge. Regarding mTOR inhibition by rapamycin, the recent study has indicated that phosphorylation of p70S6K is significantly impaired by rapamycin in HDM-challenged mice [17]. Consistent with this, our data have revealed that treatment with rapamycin after OVA inhalation substantially diminishes the phosphorylation of mTOR and p70S6K as well as the increases in HIF-1α activity and VEGF levels. These observations encourage the view that mTOR signaling is required for the induction of HIF-1α activity and VEGF expression. Similarly, mechanical strain of airway smooth muscle induces HIF-1α-dependent VEGF expression via mTOR pathways [55]. Further, although not yet fully established in allergic inflammation, several studies have documented the vital roles of the mTOR/HIF-1α/VEGF pathway in a variety of cellular conditions, including malignancy and angiogenesis [56]–[58]. Altogether, it is proposed that activation of mTOR/p70S6K may be a specific modulator for the induction of HIF-1α and its target gene VEGF in allergic airway disease (mTOR/HIF-1α/VEGF axis).

We next determined whether PI3K/Akt participates in activation of mTOR by OVA inhalation. As described earlier, PI3K/Akt pathway is known to upregulate HIF-1α and VEGF expression in an mTOR-dependent manner [57], [58]. In line with these findings, treatment with a PI3K inhibitor, LY294002 inhibits the phosphorylation of mTOR and p70S6K in an OVA-induced asthma model. Additionally, the increased levels of HIF-1α activity and VEGF expression as well as typical asthmatic symptoms after OVA inhalation are substantially attenuated by LY294002. Accordingly, PI3K/Akt is suggested to act upstream of the mTOR/HIF-1α/VEGF signaling pathway.

Much progress has been accomplished in understanding the functions of PKCs in allergic airway disorders. As stated above, PKC δ, a member of the novel PKC subfamily, has been known to be implicated in AHR, allergic inflammation, and eosinophil migration observed in asthma [10]–[12]. Intriguingly, several lines of evidence indicate that mTOR-mediated signaling can occur through upregulation of PKC δ activity in various cell types. For example, in endothelial cells, PKC δ has been shown to activate mTOR, modulating NF-κB activation and ICAM-1 expression [25]. PKC δ could also be necessary for insulin-stimulated mTOR and p70S6K phosphorylation in cardiac myocytes [38]. Thus, the activation of mTOR by PKC δ prompted us to investigate the functional importance of this relationship in murine models of asthma. As expected, levels of p-PKC δ in lung tissues are significantly increased after OVA inhalation compared with those after saline inhalation, implying PKC δ activation in allergic inflammatory response. The augmented levels of p-PKC δ are markedly reduced by rottlerin. Subsequent study has shown that rottlerin represses the phosphorylation of PI3K, Akt, mTOR, and p70S6K, which parallels with the loss of HIF-1α activity and VEGF levels. Therefore, the inhibition of PKC δ by rottlerin is supposed to attenuate AHR and airway inflammation through suppressing the PI3K/Akt/mTOR/HIF-1α/VEGF pathway in an asthma model.

Time course analysis of the activation of PKC δ and its downstream mediators may shed light on the modifier role of PKC δ in allergic airway disease. According to the current investigation, the level of p-PKC δ begins to increase significantly at 1 h after OVA inhalation, followed by p-Akt, p-mTOR, HIF-1α, and VEGF. Thus, these data provide direct evidence for the presence of the PKC δ-PI3K/Akt-mTOR-HIF-1α-VEGF sequence in an asthma model. Subsequently, to further confirm the stimulatory role of PKC δ in allergic response, an *in vitro* experiment was performed using BEAS-2B cells. Previous evidence has demonstrated that PI3K/AKT/mTOR pathway is involved in the activation of HIF-1α and VEGF by TNF-α/IL-4 in these cells [33]. Consistent with this, in the present study, TNF-α/IL-4 stimulation facilitates the levels of HIF-1α activity and VEGF expression as well as the phosphorylation of Akt and its downstream signal molecules in BEAS-2B cells. Besides, we have identified that p-PKC δ levels are markedly increased in our cell system incubated with TNF-α/IL-4. However, the enhanced levels of p-PKC δ, p-Akt, p-mTOR, p-p70S6K, HIF-1α, and VEGF after stimulation with TNF-α/IL-4 are significantly reduced by rottlerin or siRNA-PKC δ. Therefore, our findings lend weight to the contention that PKC δ modulates HIF-1α activation, therewith inducing VEGF expression through the PI3K/Akt/mTOR pathway in allergic airway disease.

Nevertheless, it is important to consider the potential limitations of the present study. Since the specificity of all chemical and genetic inhibitors may be questioned, we cannot rule out the possibility that other PKC isoforms besides PKC δ may be involved in the allergic airway response to OVA inhalation. For instance, a previous report has shown that rottlerin has no effect on PKC δ activity, but instead inhibits a number of other kinases [59]. However, in our study, rottlerin substantially attenuates PKC δ activity in OVA-challenged lungs. Moreover, in each set of *in vivo* and *in vitro* experiments, we have obtained similar results with rottlerin and siRNA-PKC δ. Even though the roles of other related PKC isozymes have not been sufficiently clarified in the present study, these data suggest that PKC δ indeed regulates allergic airway inflammation in our system.

As noted, mTOR exists in two distinct complexes, mTOR complex 1 (mTORC1) and mTOR complex 2 (mTORC2) that differ in their subunit composition and sensitivity to rapamycin. mTORC1 regulates ribosomal biogenesis and protein translation through p70S6K and 4E-BP1, whereas mTORC2, which is a downstream mediator of PI3K, upregulates Akt activity by direct phosphorylation of Ser^473^ (PI3K-mTORC2-Akt axis) [60]. Although our study does not discriminate these multi-protein complexes, it is apparent that the phosphorylation of mTORC1 is inhibited by rapamycin or upstream inhibitors in OVA-inhaled mice (as indicated by reduction in p-p70S6K). Interestingly, rapamycin is recognized as a universal inhibitor of mTORC1 and a cell-type specific inhibitor of mTORC2 [17]. Thus, the possibility that rapamycin might block mTORC2 activity as well in our model cannot be excluded. Furthermore, given the PI3K-mTORC2-Akt module as well as the fact that rottlerin or LY294002 attenuates the phosphorylation of PI3K and Akt (Ser^473^) in asthmatic mice, PI3K inhibition with these inhibitors are believed to reduce Akt signaling by downregulating mTORC2 activity. The precise mechanism of action of mTORC2 in an asthma model should be evaluated in further researches.

**Figure 13:**
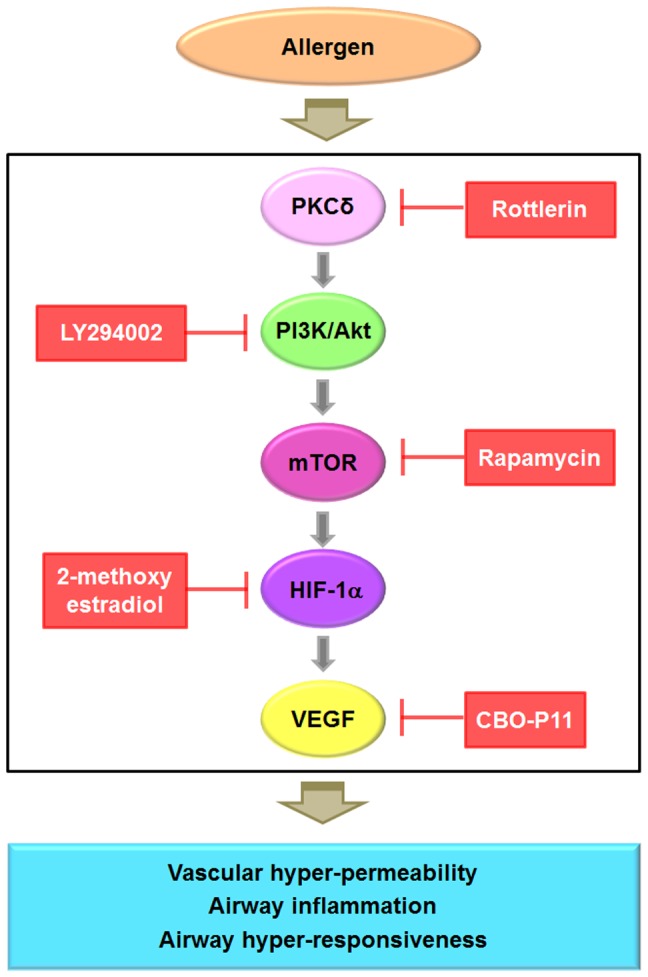
A proposed mechanism for the involvement of PKC δ in allergic asthma.

In summary, we have shown herein that treatment of allergic mice with rottlerin results in the inhibition of PKC δ and the subsequent disruption of a PI3K/Akt/mTOR/HIF-1α/VEGF module, thereby reversing all pathophysiologic symptoms of asthma. These data suggest that PKC δ is a positive regulator of PI3K/Akt/mTOR/HIF-1α/VEGF pathway (Fig. 13). As far as we know, the present report is the first to describe the involvement of this signal transduction cascade in allergic airway disease. In conclusion, PKC δ could be a key mediator of allergic airway inflammation and a promising target for therapeutic intervention.
